# Chromosome Painting Facilitates Anchoring Reference Genome Sequence to Chromosomes *In Situ* and Integrated Karyotyping in Banana (*Musa* Spp.)

**DOI:** 10.3389/fpls.2019.01503

**Published:** 2019-11-20

**Authors:** Denisa Šimoníková, Alžbeěta Němečková, Miroslava Karafiátová, Brigitte Uwimana, Rony Swennen, Jaroslav Doležel, Eva Hřibová

**Affiliations:** ^1^Institute of Experimental Botany, Czech Academy of Sciences, Centre of the Region Hana for Biotechnological and Agricultural Research, Olomouc, Czechia; ^2^Banana Breeding, International Institute of Tropical Agriculture, Kampala, Uganda; ^3^Bioversity International, Banana Genetic Resources, Heverlee, Belgium; ^4^Division of Crop Biotechnics, Laboratory of Tropical Crop Improvement, Katholieke Universiteit Leuven, Leuven, Belgium; ^5^Banana Breeding, International Institute of Tropical Agriculture, Arusha, Tanzania

**Keywords:** banana, chromosome identification, fluorescence *in situ* hybridization, molecular karyotype, *Musa*, oligo painting FISH

## Abstract

Oligo painting FISH was established to identify all chromosomes in banana (*Musa* spp.) and to anchor pseudomolecules of reference genome sequence of *Musa acuminata* spp. *malaccensis* “DH Pahang” to individual chromosomes *in situ*. A total of 19 chromosome/chromosome-arm specific oligo painting probes were developed and were shown to be suitable for molecular cytogenetic studies in genus *Musa*. For the first time, molecular karyotypes of diploid *M. acuminata* spp. *malaccensis* (A genome), *M. balbisiana* (B genome), and *M. schizocarpa* (S genome) from the Eumusa section of *Musa*, which contributed to the evolution of edible banana cultivars, were established. This was achieved after a combined use of oligo painting probes and a set of previously developed banana cytogenetic markers. The density of oligo painting probes was sufficient to study chromosomal rearrangements on mitotic as well as on meiotic pachytene chromosomes. This advance will enable comparative FISH mapping and identification of chromosomal translocations which accompanied genome evolution and speciation in the family *Musaceae*.

## Introduction

Bananas (*Musa* spp.) are grown in tropical and subtropical regions of South East Asia, Africa and South America (Häkkinen, 2013; Janssens et al., 2016). They are one of the world’s major fruit crops, a staple and important export commodity for millions of people living mainly in developing countries. Despite the importance and breeding efforts (Ortiz and Swennen, 2014; Brown et al., 2017), little is known about banana genome structure, organization and evolution at chromosomal level across the whole *Musaceae* family.

The genus *Musa* comprises about 75 species and numerous cultivated edible clones. Based on a set of morphological descriptors (IPGRI-INIBAP/CIRAD, 1996) and basic chromosome number (x), the genus *Musa* has traditionally been divided into four sections: Eumusa (x = 11), Rhodochlamys (x = 11), Australimusa (x = 10), and Callimusa (x = 9, 10) (Cheesman, 1947). Argent (1976) created a separate section Ingentimusa which contains a single species *Musa ingens* with the lowest basic chromosome number (x = 7). However, genotyping using molecular markers revealed close relationship of *M. ingens* with other species of sections Callimusa and Australimusa (Li et al., 2010). Most of the modern edible banana clones originated within section Eumusa after intra- and inter-specific crosses between two wild diploid species *M. acuminata* (donor of A genome) and *M. balbisiana* (donor of B genome). In some cases, diploid *M. schizocarpa* (S genome) contributed to the evolution of edible clones, mainly after cross-breeding with diploid *M. acuminata* (Carreel et al., 1994; Čížková et al., 2013; Němečková et al., 2018). The spontaneous intra- and inter-specific crosses gave arise to seed sterile diploid (AA, AB) and triploid (AAA, AAB, or ABB) edible banana cultivars. Although tetraploid clones (AAAB, AABB) that originated spontaneously are known (Simmonds and Shepherd, 1955; Simmonds, 1956), currently cultivated tetraploid bananas were obtained in the breeding programs.

Species of genus *Musa* have a relatively small genome, ranging from 550 to 750 Mbp/1C (Doležel et al., 1994; Lysák et al., 1999; Asif et al., 2001; Kamaté et al., 2001; Bartoš et al., 2005; Čížková et al., 2015) and until now it was possible to identify only a few chromosomes in their karyotypes. The attempts were hampered by the relatively high number of chromosomes, their small size at mitotic metaphase (1–2 µm) and morphological similarity (Doleželová et al., 1998; Osuji et al., 1998; D’Hont et al., 2000). Chromosome banding, which was found informative in plant species with large and repeat-rich genomes, including wheat and rye (Gill and Kimber, 1977; Gill et al., 1991), did not result in diagnostic chromosome banding patterns in *Musa*, similar to many other plant species (Greilhuber, 1977; Schubert et al., 2001).

The application of fluorescence *in situ* hybridization (FISH), usually done with probes for DNA repeats with chromosome-specific distribution, provided a powerful approach to identify chromosomes in a range of plant species and study chromosome structural changes (e.g., Liu et al., 2011; Danilova et al., 2014; Amosova et al., 2017; Hou et al., 2018). Unfortunately, its use in *Musa* was hampered by the lack of suitable probes (Doleželová et al., 1998; Osuji et al., 1998; Valárik et al., 2002; Hřibová et al., 2007; Čížková et al., 2013). Until now, only NOR-bearing satellite chromosome, two chromosomes with clusters of tandem repeats CL18 and CL33, and two chromosomes bearing 5S rDNA loci can be cytogenetically identified in *M. acuminata* and *M. balbisiana* (Čížková et al., 2013). In *M. schizocarpa*, one chromosome pair bearing NOR and two chromosome pairs bearing tandem repeats CL18 and CL33 and other four chromosome pairs with 5S rDNA loci can be identified cytogenetically (Čížková et al., 2013). Even the mining of the reference genome sequence of *M. acuminata* “DH Pahang” (D’Hont et al., 2012) did not result in identification of sequences suitable as FISH probes useful for unambiguous identification of all *Musa* chromosomes and their anchoring to the genome sequence.

A method for chromosome painting, which allows fluorescent labeling of whole chromosomes, was developed in the late 1980s. This advance revolutionized human cytogenetics and found numerous applications in animal cytogenetics (e.g., Speicher et al., 1996; Cremer and Cremer, 2001; Ferguson-Smith and Trifonov, 2007). The original method was based on FISH with whole chromosome probes obtained from chromosomes isolated by flow cytometric sorting or microdissection. This was the reason why the method failed in plants where a majority of DNA repeats is distributed across the whole genome and only a minority of sequences are unique and chromosome-specific (Schubert et al., 2001). A solution was to use pools of chromosome-specific BAC (Bacterial Artificial Chromosome) clones (Lysák et al., 2001). However, the development of chromosome BAC pools requires whole genome sequence obtained after clone by clone (BAC by BAC) sequencing to identify single or low copy BAC clones useful for painting. Thus, the method is suitable for species with small genomes and containing low amounts of DNA repeats. Till now, painting using chromosome-specific BAC pools was used in dicotyledonous species with small nuclear genomes— *Arabidopsis* and its closely related species (e.g., Lysák et al., 2001; Mandáková and Lysák, 2008; Mandáková et al., 2013) as well as in monocot *Brachypodium distachyon* (Idziak et al., 2014). The attempts to use BAC FISH in banana were not successful due to the lack of a larger number of BAC clones containing single or low copy sequences (Hřibová et al., 2008).

The recent progress in the production of reference genome sequences and in technologies for DNA synthesis provided an alternative opportunity for affordable preparation of whole chromosome probes (chromosome paints) for FISH. The method called oligo painting FISH (Han et al., 2015) is based on *in silico* identification of large numbers of short (usually 45–50 bp) and unique (single copy) sequences in pseudomolecules of individual chromosomes, or their parts, synthesis of oligonucleotides, and their fluorescent labeling. A pool of synthesized and fluorescently labeled oligonucleotides is then used as a probe for FISH. Thus, the oligo painting FISH provides an opportunity to identify individual chromosomes and chromosome regions in *Musa*, perform comparative chromosome analysis and characterize chromosomal rearrangements (Qu et al., 2017; Braz et al., 2018; Xin et al., 2018; Jiang, 2019).

The present study fills the important gap in molecular cytogenetics of *Musa*. The application of oligo painting FISH described here allows anchoring genome sequence to chromosomes *in situ* and unambiguous identification of all *Musa* chromosomes after development of molecular karyotypes by a combined use of oligo painting probes and existing cytogenetic landmarks. Molecular karyotypes are described and compared for the three main genomes of Eumusa section—*M. acuminata* ssp. *malaccensis*, *M. balbisiana*, and *M. schizocarpa*, which contributed to the evolution of many edible banana clones.

## Materials and Methods

### Plant Material and Preparations of Chromosome Spreads

Representatives of three species from the section Eumusa were obtained as *in vitro* rooted plants from the International *Musa* Transit Centre (ITC, Bioversity International, Leuven, Belgium). *In vitro* plants were transferred to garden soil and maintained in a heated greenhouse. **Table 1** lists the accessions used in this study. Male buds of *M. acuminata* “Pahang” and *M. balbisiana* “Tani” were obtained from the research station of the International Institute of Tropical Agriculture in Sendusu, Uganda.

**Table 1:** List of analyzed accessions, their genomic constitution, genome size, and the number of loci identified on mitotic metaphase chromosomes (data from Čížková et al., 2013).

Species	Accession name	ITC code^a^	Genomic constitution	Genome size (1C)	Chromosome number (2n)	The number of loci in diploid cells (2n = 22)				
						45S rDNA	5S rDNA	BAC 2G17	CL33	CL18
*M. acuminata* ssp. *malaccensis*	Pahang	0609	AA	594 Mbp**^b^**	22	2	6	2	4	2
*M. balbisiana*	Tani	1120	BB	551 Mbp**^b^**	22	2	6	2	0	4
*M. schizocarpa*	Schizocarpa	0560	SS	671 Mbp**^b^**	22	2	12	2	4	2

a Code assigned by the International Transit Centre (ITC, Leuven, Belgium)

b DNA content was estimated by flow cytometry using Glycine max L. cv. Polanka (2C = 2.5pg DNA) which served as an internal reference standard (Čížková et al., 2013).

Actively growing root tips (∼1 cm long) were collected into 50-mM phosphate buffer (pH 7.0) containing 0.2% (v/v) β-mercaptoethanol, pre-treated in 0.05% (w/v) 8-hydroxyquinoline for three hours at room temperature, fixed in 3:1 ethanol:acetic acid fixative overnight, and stored in 70% ethanol. Preparation of protoplast suspensions was performed according to Doležel et al. (1998). Briefly, after digesting root tip segments in a mixture of 2% (w/v) cellulase and 2% (w/v) pectinase in 75-mM KCl and 7.5-mM EDTA (pH 4) for 90 min at 30°C, the suspension of resulting protoplasts was filtered through a 150-µm nylon mesh, pelleted, and washed in 70% ethanol. For further use, the protoplast suspension was stored in 70% ethanol at −20°C. Mitotic metaphase chromosome spreads were prepared by dropping method according to Doležel et al. (1998), the slides were postfixed in 4% (v/v) formaldehyde solution in 2x SSC solution and used for FISH.

Preparation of pachytene chromosome spreads was performed according to Mandáková and Lysák (2008), with minor modifications. Male flowers were fixed in 3:1 ethanol:acetic acid fixative overnight and stored in 70% ethanol at −20°C. Anthers were incubated in 0.3% (w/v) mix of cellulase, cytohelicase, and pectolyase (Sigma Aldrich, Darmstadt, Germany) for 30 min at 37°C. After the incubation in the enzyme mixture, the anthers were dissected in a drop of 60% (v/v) acetic acid on a microscopic slide and spread on the slide placed on a metal hot plate (50°C) after adding 60% (v/v) acetic acid for 25 s. The preparations were fixed in 3:1 ethanol:acetic acid fixative, air-dried, and used for FISH.

### Identification of Specific Oligomers and Labeling of the Oligo Probes

Oligomers specific for individual chromosome arms were identified in the reference genome sequence of *M. acuminata* “DH Pahang” v.2 (Martin et al., 2016) using Chorus pipeline (Han et al., 2015). Sets of 20,000 oligomers (45-mers) per one library were synthesized by Arbor Biosciences (Ann Arbor, Michigan, USA). Labeled oligomer probes were prepared according to Han et al. (2015). Briefly, the oligomer libraries were amplified using emulsion PCR (Murgha et al., 2014), where F primer contained T7 RNA polymerase promoter. The emulsified PCR product was washed with water-saturated diethyl ether and ethyl acetate and purified with QIAquick PCR purification kit (Qiagen, Hilden, Germany). The product (480 ng DNA) was used for T7 *in vitro* transcription with MEGAshortscript T7 Kit (ThermoFisher Scientific/Invitrogen, Waltham, Massachusetts, USA) at 37°C for 4 h. The RNA product was purified using RNeasy Mini Kit (Qiagen) and 42 µg of RNA was reverse-transcribed with either digoxigenin-, biotin-, or CY5-labeled R primer (Eurofins Genomics, Ebersberg, Germany) using Superscript II Reverse Transcriptase and SUPERase-In RNase inhibitor (ThermoFisher Scientific/Invitrogen). The RNA : DNA hybrids were cleaned with Quick-RNA MiniPrep Kit (Zymo Research, Freiburg im Breisgau, Germany) and hydrolyzed with RNase H (New England Biolabs, Ipswich, Massachusetts, USA) and finally with RNase A (ThermoFisher Scientific/Invitrogen). The products were purified with Quick-RNA MiniPrep Kit (Zymo Research) and eluted with nuclease-free water to obtain single-stranded labeled oligomers, which were used as FISH probes.

### Preparation of Other Cytogenetic Markers for FISH

Probes specific for ribosomal DNA sequences were prepared by labeling *Radka1* (part of 26S rRNA gene) and *Radka2* (contains 5S rRNA gene and non-transcribed spacer) DNA clones (Valárik et al., 2002) with biotin-16-dUTP (Roche Applied Science, Penzberg, Germany) or aminoallyl-dUTP-CY5 (Jena Biosciences, Jena, Germany) by PCR using T3 (forward) and T7 (reverse) primers (Invitrogen). Probes for tandem repeats CL18 and CL33 (Hřibová et al., 2010) were amplified using specific primers and labeled with aminoallyl-dUTP-CY5 or fluorescein-12-dUTP (Jena Biosciences, Jena, Germany) by PCR according to Čížková et al. (2013). Single copy BAC clone 2G17 (Hřibová et al., 2008) was labeled by digoxigenin-11-dUTP nick translation following manufacturer’s recommendation (Roche Applied Science, Penzberg, Germany).

### Fluorescence *In Situ* Hybridization and Image Analysis

Hybridization mix (30 µl) containing 50% (v/v) formamide, 10% (w/v) dextran sulfate in 2x SSC and 10 ng/µl of labeled probe was added onto slide and denatured for 3 min at 80°C. Hybridization was carried out overnight at 37°C. The sites of hybridization of digoxigenin- and biotin-labeled probes were detected using anti-digoxigenin-FITC (Roche Applied Science) and streptavidin-Cy3 (ThermoFisher Scientific/Invitrogen), respectively. Chromosomes were counterstained with DAPI and mounted in VECTASHIELD Antifade Mounting Medium (Vector Laboratories, Burlingame, CA, USA). The slides were examined with Axio Imager Z.2 Zeiss microscope (Zeiss, Oberkochen, Germany) equipped with Cool Cube 1 camera (Metasystems, Altlussheim, Germany) and appropriate optical filters. The capture of fluorescence signals, merging the layers, and measurement of chromosome length were performed with ISIS software 5.4.7 (Metasystems), the final image adjustment and creation of idiograms were done in Adobe Photoshop CS5.

## Results

### Development of Chromosome Painting Probes and *In Situ* Hybridization

In order to produce chromosome arm-specific painting probes, unique *k*- mers were identified according to Han et al. (2015) in the reference genome sequence of the doubled haploid banana (*M. acuminata* “DH Pahang”; Martin et al., 2016) and analyzed with the Chorus program (https://github.com/forrestzhang/Chorus). While eight pseudomolecules corresponded to metacentric chromosomes, pseudomolecules 1, 2, and 10 appeared to be acrocentric with peri-centromeric region occupying an entire chromosome arm. The density of unique oligomers was lower in peri-centromeric regions in all pseudomolecules (**Supplementary Figure S1**) and these regions were excluded from the selection of oligomers for painting probes. The number of unique oligomers ranged from 79,896 to 127,835 for pseudomolecules 2 and 6, respectively. Sets of 20,000 45-mers specific to individual chromosome arms were then selected in Chorus, synthesized as so called immortal libraries and labeled directly by Cy5 or indirectly by biotin or digoxigenin as described in Materials and Methods. Oligomer libraries were designed to achieve a density of 0.9 to 2.1 oligomers per 1-kb chromosome sequence (**Supplementary Table S1**). To confirm that it is not possible to paint peri-centromeric regions with low oligomer densities and large gaps between low copy oligomers, a painting probe was prepared from peri-centromeric region of pseudomolecule 3. In total, 8,317 oligomers spanning this region (∼10.5 Mb long) ensured an average density of ∼0.8 oligomers/kb.

**Figure 1:**
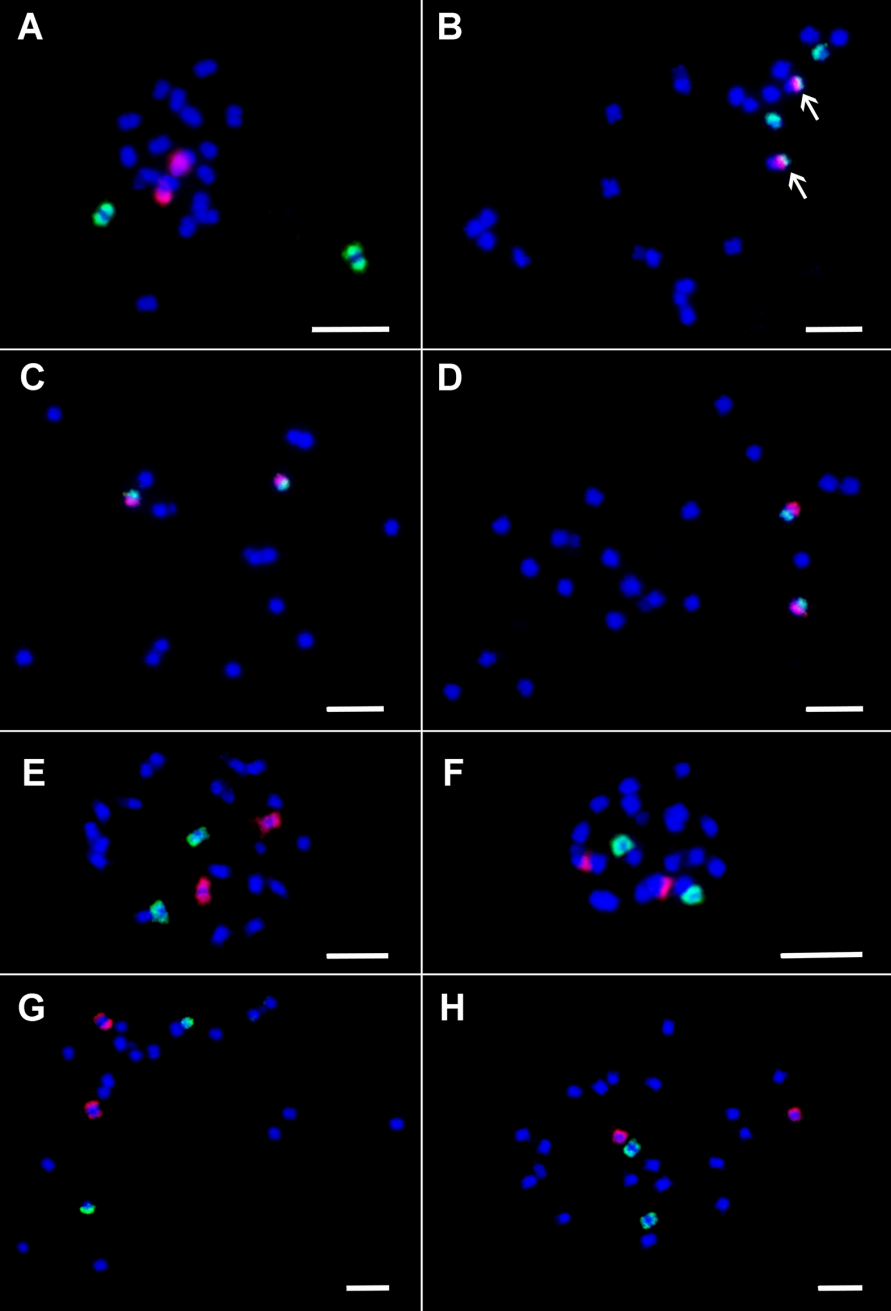
Oligo painting FISH on mitotic metaphase plates of three species of *Musa*. **(A)** *M. acuminata* ssp. *malaccensis* “Pahang” (2n = 22, AA; chromosome 1 in red, chromosome 3 in green). **(B)** *M. balbisiana* “Tani” (2n = 22, BB; chromosome 1 in red, chromosome 3 in green). **(C)** *M. acuminata* ssp. *malaccensis* “Pahang” (2n = 22, AA; short arm of chromosome 4 in green, its long arm in red). **(D)** *M. acuminata* ssp. *malaccensis* “Pahang” (2n = 22, AA; short arm of chromosome 5 in red, its long arm in green). **(E)** *M. acuminata* ssp. *malaccensis* “Pahang” (2n = 22, AA; chromosome 6 in red, chromosome 7 in green. **(F)** *M. acuminata* ssp. *malaccensis* “Pahang” (2n = 22, AA; chromosome 10 in red, chromosome 11 in green. **(G)** *Musa schizocarpa* “Schizocarpa” (2n = 2x = 22, SS; chromosome 8 in red, chromosome 2 in green). **(H)** *Musa schizocarpa* “Schizocarpa” (2n = 2x = 22, SS; chromosome 11 in red, chromosome 9 in green). Chromosomes were counterstained with DAPI (blue). Arrows point to the region of chromosome 3 translocated to chromosome 1 in *M. balbisiana*. Bars = 5 µm.

First, the painting probes were hybridized to mitotic metaphase chromosomes spreads of *M. acuminata* ssp. *malaccensis* (A genome)—the genotype from which the *Musa* reference genome sequence was developed. FISH with the painting probes resulted in visible signals covering chromosome arms along their lengths (**Figures 1A–F**). This observation confirmed that the probes had the expected parameters. Moreover, because the painting highlighted individual chromosome arms, it was possible to anchor pseudomolecules to individual chromosome arms. This work revealed that in the assembly, pseudomolecules 1, 6, 7 start with long arms and end with short arms, i.e., they are oriented inversely to the way karyotypes are presented, where the short arm of the chromosome is on top and the long arm on the bottom.

Following this, the painting probes were used for FISH in *M. balbisiana* (B genome) and *M. schizocarpa* (S genome) (**Figures 1B, G, H**). Comparison of chromosome and/or chromosome-arm painting in *M. acuminata* ssp. *malaccensis* and *M. schizocarpa* did not reveal any large chromosome translocations differentiating both species. On the other hand, a large translocation of the long arm of chromosome 3 to long (painted) arm of chromosome 1 was found in *M. balbisiana* (**Figure 1B**).

**Figure 2:**
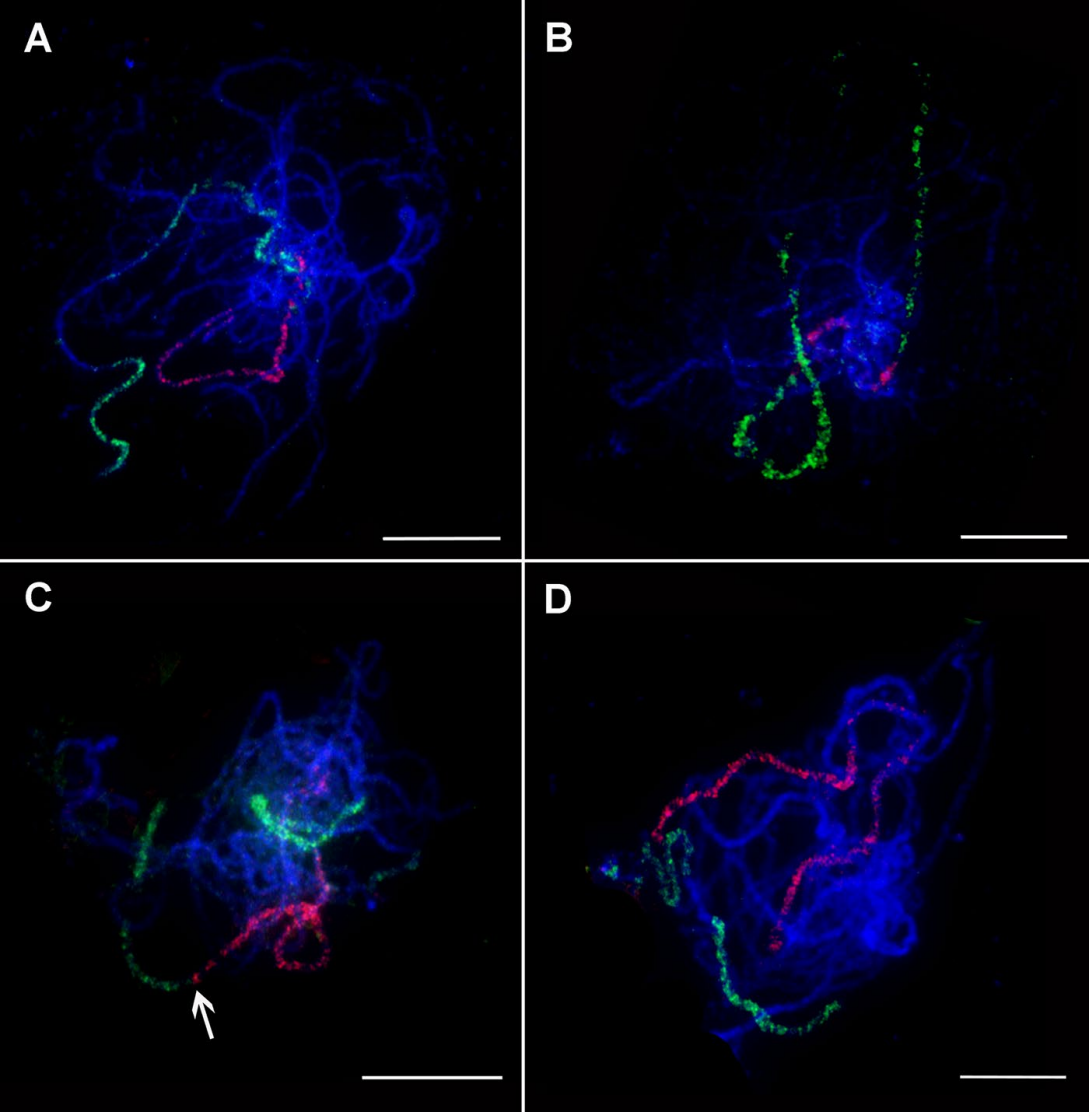
Oligo painting FISH on meiotic pachytene chromosome spreads of *Musa*. **(A)** *M. acuminata* ssp. *malaccensis* “Pahang” (2n = 22, AA; chromosome 1 in red, chromosome 4 in green). **(B)** *M. acuminata* ssp. *malaccensis* “Pahang” (2n = 22, AA; (peri-)centromeric region in red, chromosome 3 in green). **(C)** *M. balbisiana* “Tani” (2n = 22, BB; chromosome 1 in red, chromosome 3 in green). **(D)** *M. balbisiana* “Tani” (2n = 22, BB; chromosome 5 in red, chromosome 11 in green). Chromosomes were counterstained with DAPI (blue). Arrows point to the region translocated from chromosome 3 to chromosome 1 in *M. balbisiana*. Bars = 10 µm.

The small size of condensed mitotic metaphase chromosomes reduces the longitudinal resolution of chromosome painting and hence a chance to discover small structural rearrangements. An alternative is to perform chromosome painting with meiotic pachytene chromosomes (**Figure 2**) which are approximately fifty times longer. When hybridized to pachytene chromosome spreads of *M. acuminata* ssp. *malaccensis*, painting probes provided visible signals and the opportunity to analyze chromosome structure in more detail. This experiment showed that banana chromosomes do not contain large blocks of heterochromatin in distal and subtelomeric regions (**Figure 2**). Taking the advantage of higher spatial resolution, the set of oligo painting probes developed in this work will be suitable to visualize meiotic processes such as crossing over and synapsis. Following this, pachytene chromosome spreads of *M. acuminata* ssp. *malaccensis* were used to evaluate the signal of peri-centromeric painting probe designed for chromosome 3. FISH with the probe did not result in a continuous signal along the whole region. Instead, discontinuous signals, with signal-free gaps along most of the (peri-)centromeric region of chromosome 3 (**Figure 2B**), were observed. Based on this observation, painting probes were not designed for (peri-)centromeric regions of the remaining ten banana chromosomes.

### Integration of Cytogenetic Landmarks and Oligopaints

In order to utilize the existing probes for FISH in *Musa* and develop a highly informative toolbox to characterize *Musa* chromosome structure, the existing cytogenetic landmarks were integrated with the painting probes.

**Figure 3:**
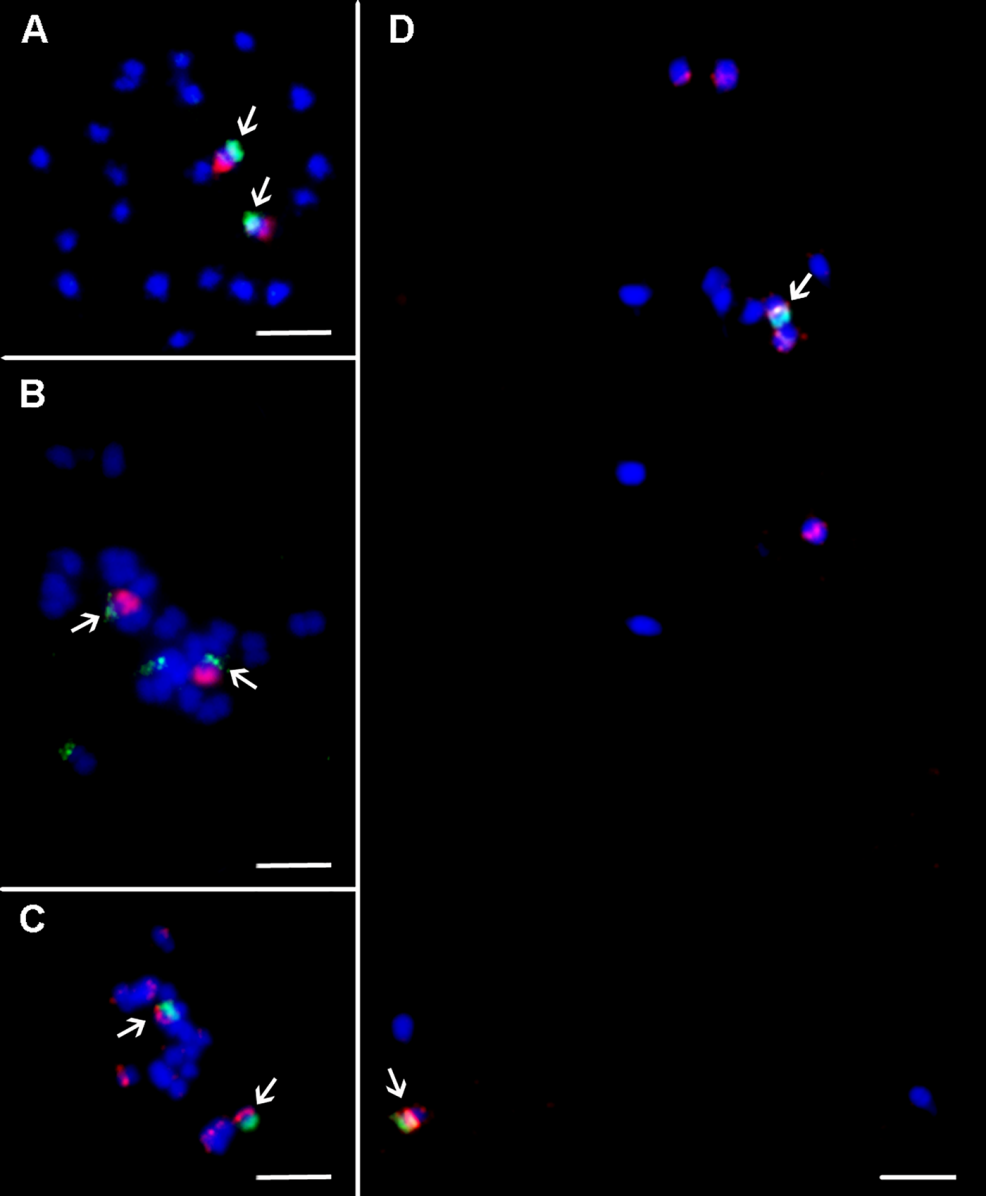
Integration of oligo painting FISH and existing cytogenetic markers on mitotic metaphase plates of *Musa*. **(A)** *M. acuminata* ssp. *malaccensis* “Pahang” (2n = 22, AA; chromosome 1 in red, BAC clone 2G17 in green). **(B)** *M. schizocarpa* “Schizocarpa” (2n = 22, SS; chromosome 2 in red, tandem repeat CL33 in green). **(C)** *M. schizocarpa* “Schizocarpa” (2n = 22, SS; short arm of chromosome 4 in green, 5S rRNA in red—two loci are localized on long arm of chromosome 4). **(D)** *M. acuminata* ssp. *malaccensis* “Pahang” (2n = 22, AA; 5S rRNA in red, short arm of chromosome 3 in green bears 5S rRNA). Chromosomes were counterstained with DAPI (blue). Bars = 5 µm. Arrows indicate colocalization of oligo painting FISH probes with existing cytogenetic markers.

**Figure 4:**
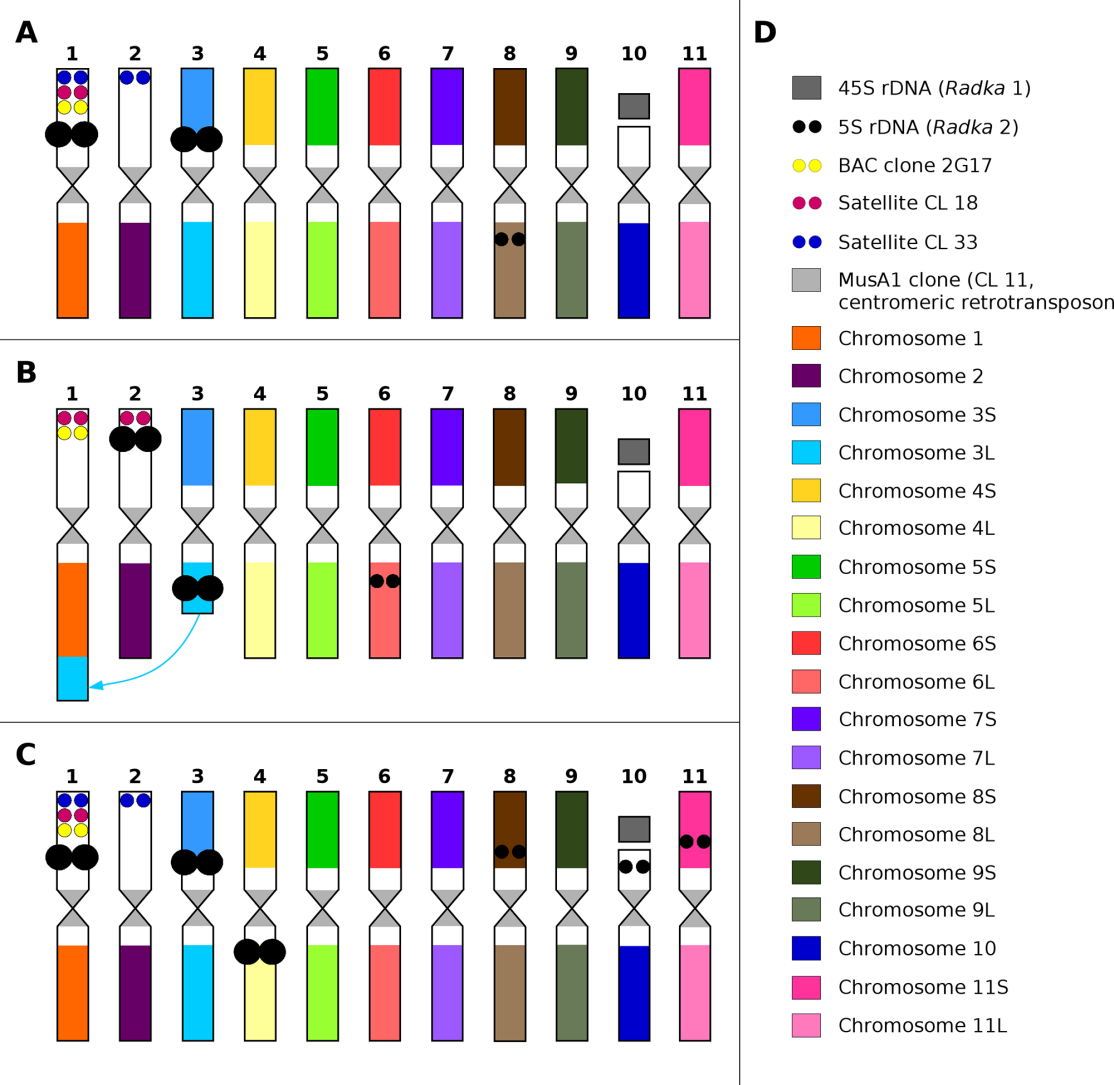
Idiograms of three diploid species of Eumusa section of *Musa*. **(A)** *Musa acuminata* ssp. *malaccensis* “Pahang” (genome A) ITC 0609. **(B)** *Musa balbisiana* “Tani” (genome B) ITC 1120. **(C)** *Musa schizocarpa* “Schizocarpa” (genome S) ITC 0560. **(D)** multicolored scheme.

45S rRNA genes mapped to secondary constriction located on non-painted arm of chromosome 10 in all three *Musa* species. The probe for 5S rRNA genes localized to different chromosome regions and on different chromosomes in the three *Musa* species studied. In *M. acuminata* ssp. *malaccensis*, six signals of 5S rDNA were observed on mitotic metaphase plates and were localized in sub-telomeric region of chromosome 1 and long arm of chromosome 8, and in peri-centromeric region on short arm of chromosome 3. Six hybridization signals with 5S rDNA probe were observed also in mitotic metaphase plate of *M. balbisiana*. Two pairs of strong signals were localized in sub-telomeric region of chromosome 2 (non-painted arm) and in peri-centromeric region of the long arm of chromosome 3. Additional weak signal was observed in peri-centromeric region of the long arm of chromosome 6 (**Figures 3** and **4**). In *M. schizocarpa*, three pairs of strong signals and three pairs of weaker signals were observed after FISH with 5S rDNA probe on mitotic metaphase plate. Sub-telomeric region of chromosome 1 (non-painted arm) and peri-centromeric region of short arm of chromosome 3 and long arm of chromosome 4 contained strong signals of 5S rDNA. Additional weaker signals of 5S rDNA probe were observed in peri-centromeric regions of short arm of chromosome 8 and short arm of chromosome 11, as well as on the non-painted arm of chromosome 10 (**Figures 3** and **4**).

Tandem organized repeats CL18, CL33, and BAC clone 2G17 were localized on non-painted arm of chromosome 1 in all three *Musa* species, except satellite CL33, which was not detected on any chromosome in *M. balbisiana*. In contrast, additional signal of tandem repeat CL33 located on non-painted arm of chromosome 2 was observed in *M. acuminata* ssp. *malaccensis* and in *M. schizocarpa* (**Figures 3** and **4**). Finally, additional signal of tandem repeat CL18 was observed in *M. balbisiana* on the non-painted arm of chromosome 2.

## Discussion

Until recently, chromosome painting could be used only in plants whose genomes were sequenced clone by clone (The Arabidopsis Genome Initiative, 2000; The International Brachypodium Initiative, 2010) and chromosome painting was achieved by FISH with pools of single copy BAC clones that covered entire chromosomes. Importantly, chromosome paints developed in one species could be used in related species, providing a powerful approach for comparative karyotype analysis and for tracing karyotype changes during the evolution and speciation (e.g., Lysák et al., 2001; Idziak et al., 2014). Unfortunately, this painting method cannot be used in species with large genomes due to the prevalence of repetitive DNA and in species not closely related to those for which painting using BAC pools was developed.

The progress in DNA sequencing technology and assembly algorithms resulted in a shift from the clone by clone sequencing to shotgun sequencing and a majority of plant genomes has been sequenced in this way (Hamilton and Buell, 2012; Zimin et al., 2017; Belser et al., 2018). The availability of reference genome sequences and the affordable cost of synthesizing short oligonucleotides offered a direct way to develop chromosome paints (Han et al., 2015). Here, thousands of short single copy sequences are identified, bulk synthesized, fluorescently labeled and used as probes for FISH (Han et al., 2015). Pools of labeled oligonucleotide probes were found suitable for FISH on somatic metaphase chromosomes, meiotic pachytene chromosomes and interphase nuclei (Han et al., 2015; Filiault et al., 2018; Xin et al., 2018; Albert et al., 2019; Jiang, 2019). Successful applications of oligo painting FISH include construction of molecular cytogenetic karyotypes (Braz et al., 2018; Qu et al., 2017; Meng et al., 2018), identification of large chromosomal rearrangements, analysis of chromosome pairing in meiosis (Han et al., 2015; He et al., 2018; Albert et al., 2019), as well as the visualization of the arrangement of chromosomes in 3D space of interphase nuclei (Albert et al., 2019).

Despite the availability of a reference genome sequence of *M. acuminata ssp. malaccensis* (D’Hont et al., 2012; Martin et al., 2016) and resequencing of more than 120 accessions of *Musa* (Dupouy et al., 2019), DNA pseudomolecules have not been anchored to individual chromosomes and molecular karyotype of *Musa* has not been developed to date. In many plant species, tandem organized repeats serve as useful probes for FISH to identify individual chromosomes and their regions (Hřibová et al., 2007; Badaeva et al., 2015; Koo et al., 2016; Křivánková et al., 2017; Said et al., 2018). The nuclear genome of *Musa* species is relatively small (1C ∼ 500–750 Mb; Doležel et al., 1994; Lysák et al., 1999; Asif et al., 2001; Kamaté et al., 2001; Bartoš et al., 2005; Čížková et al., 2015) and until now, only a few tandem organized repeats and rDNA sequences were successfully used as cytogenetic landmarks (Balint-Kurti et al., 2000; Valárik et al., 2002; Hřibová et al., 2007; Hřibová et al., 2010; Čížková et al., 2013; Novák et al., 2014). Moreover, only one BAC clone has been used as a cytogenetic marker in *Musa* (Hřibová et al., 2008) and only four BAC clones were localized on pachytene chromosomes (De Capdeville et al., 2009). Thus, the attempts to use of BAC clones for anchoring pseudomolecules to chromosomes in banana as has been done in other species (Jiang et al., 1995; Lapitan et al., 1997; Kim et al., 2002; Idziak et al., 2014), were not successful.

Unlike the previous approaches, chromosome painting using pools of single copy oligomers offers the opportunity to establish a molecular karyotype of *Musa*, making it possible to identify individual chromosomes, follow their behavior during somatic cell cycle and meiosis, perform comparative karyotype analysis, and identify structural chromosome changes. FISH with oligo painting probes developed in this work resulted in visible hybridization signals along chromosomal arms on condensed mitotic metaphase chromosomes (**Figure 1**) as well as on less condensed pachytene chromosomes (**Figure 2**) confirming their usefulness as painting probes in *Musa*. Only small regions on pachytene chromosomes were free of painting signals. This could be either due to the presence of heterochromatin blocks, or due to gaps in the genome sequence (**Figure 2**). In contrast to chromosome arms, peri-centromeric regions were not labeled. These regions contain large gaps in the genome sequence and large proportion of repetitive DNA sequences in peri-centromeric regions (Hřibová et al., 2010; Neumann et al., 2011; D’Hont et al., 2012; Martin et al., 2016).

We demonstrate that chromosome/chromosome-arm specific oligo painting libraries designed for *M. acuminata* ssp. *malaccensis* can be used for cytogenetic analysis of related species *M. balbisiana* and *M. schizocarpa*, which played an important role in the evolution of many edible banana clones (Carreel et al., 1994; D’Hont et al., 2012; Davey et al., 2013; Čížková et al., 2013). This observation provided an opportunity for comparative karyotype analysis and identification of putative chromosome translocations. In our study, we observed translocation of long arm of chromosome 3 to long arm of chromosome 1 in *M. balbisiana* (B genome) (**Figures 1B** and **2C**). This observation confirms the result of Baurens et al. (2019), which were obtained after anchoring a dense genetic map of *M. balbisiana* “Pisang Klutuk Wulung” to *M. acuminata* ssp. *malaccensis* reference genome sequence (Martin et al., 2016). The authors estimated the size of the translocated region of long arm of chromosome 3 to be ∼8 Mb, confirming the sensitivity of oligo chromosome painting.

Co-localization of chromosome painting probes with cytogenetic markers developed earlier for *Musa* (Valárik et al., 2002; Hřibová et al., 2010; Čížková et al., 2013) offered an opportunity to create molecular karyotypes suitable for comparative analysis. The presence of 5S rRNA genes on non-collinear chromosomes in the A, B, and S genomes of *Musa* as described here indicates small chromosomal rearrangements which occurred during *Musa* speciation. On the other hand, the location of tandem organized repeats CL18, CL33, and BAC clone 2G17 on collinear chromosome arms in all three species indicates their structural homology of the chromosome arms. These observations imply that chromosomes containing a particular DNA sequence, e.g., 5S rDNA, cannot be considered as collinear. This shows a potential weakness of comparative karyotype analysis of using only a few cytogenetic markers (Fukui et al., 1994; Murata et al., 1997).

Tandem organized repeats CL18 and CL33 (Hřibová et al., 2010) were located together with 5S rRNA genes on short arms of chromosomes 1 and 2, which lacked oligopainting signals. Genome sequence of *M. acuminata* ssp. *malaccensis* includes three pseudomolecules which are represented by two large regions differing in DNA repeat composition and in density of unique oligomers (**Supplementary Figure 1**, Martin et al., 2016). The constitution of banana pseudomolecules 1, 2, and 10 indicates that they cover only one chromosome arm and a peri-centromeric region. Painting probes created for the three pseudomolecules localized to only one chromosomal arm. One of the pseudomolecules is collinear with acrocentric chromosome 10 and bears 45S rRNA locus on its short arm. The two remaining pseudomolecules represent chromosomes 1 and 2, which seem to be meta or sub-metacentric thus could miss a large sequence region. These observations indicate that these genomic regions were not completely assembled and are missing due to the presence of a large number of various tandem organized sequences.

The improved version of *M. acuminata* “DH Pahang” reference genome sequence represents 450.7 Mbp which corresponds to ∼81% of its nuclear genome size estimated by flow cytometry (Čížková et al., 2013). In addition, the reference genome sequence contains a total of 56.6-Mbp sequences, which were not anchored to the 11 pseudomolecules. The most plausible explanation why these sequences were not included in pseudomolecules is that they represent heterochromatin regions, which are difficult to sequence. However, relatively high number of unique oligomers in unanchored scaffolds as observed in this work (**Supplementary Figure 1**) indicates that the unanchored part of the reference genome sequence contains low copy sequences from euchromatic regions. Thus, these regions were probably not anchored due to the absence of DNA markers, or they were too short to be anchored using Bionano optical mapping. The use of long-read sequencing technologies such as Oxford Nanopore in combination with optical mapping (Belser et al., 2018) should further improve the current assembly and shed light on the difficult parts of *M. acuminata* ssp. *malaccensis* genome.

## Conclusions

In this work, chromosome painting probes were developed for banana (*Musa* spp.) and used to establish molecular karyotypes for three species of *Musa* that were the parents of a majority of cultivated edible banana clones. This advance made it possible to anchor reference genome sequence of banana, *Musa acuminata* spp. *malaccensis* to individual chromosomes. The study also demonstrates the potential of oligo painting FISH for comparative karyotype analysis and identification of structural chromosome changes that accompanied the evolution and speciation in the genus *Musa*.

## Data Availability Statement

All datasets generated for this study are included in the article/**Supplementary Material**.

## Author Contributions

EH and JD conceived the experiments. DŠ, AN, and MK conducted the study and processed the data. BU and RS provided the banana materials. DŠ and EH wrote the manuscript. EH, JD, and DŠ discussed the results and contributed to manuscript writing. All authors have read and approved the final manuscript.

## Conflict of Interest

The authors declare that the research was conducted in the absence of any commercial or financial relationships that could be construed as a potential conflict of interest.

## Supplementary Material

Supplementary Figure S1 Oligomer coverage of 11 pseudomolecules (labeled **A**– **K**) and concatenated unanchored scaffolds **(L)** in the reference genome of *M. acuminata*‘DH Pahang’ (Martin et al., 2016). The oligomers (45 bp) were designed using the Chorus program (Han et al., 2015) and are depicted in black. Position and coverage of tandem repeats CL18 (pink) and CL33 (green) are also shown.

## References

[B1] Albert P. S. Zhang T. Semrau K. Rouillard J. M. Kao Y. H. Wang C. R. (2019). Whole-chromosome paints in maize reveal rearrangements, nuclear domains, and chromosomal relationships. Proc. Natl. Acad. Sci. U. S. A. 116 (5), 1679–1685. 10.1073/pnas.1813957116 30655344 PMC6358699

[B2] Amosova A. V. Bolsheva N. L. Zoshchuk S. A. Twardovska M. O. Yurkevich O. Y. Andreev I. O. (2017). (A) Comparative molecular cytogenetic characterization of seven *Deschampsia (Poaceae)* species. PloS One 12 (4), e0175760. 10.1371/journal.pone.0175760 28407010 PMC5391082

[B3] Argent G. C. G. (1976). The wild bananas of Papua New Guinea. Notes R. Bot. Gard. Edinburgh. 35 (1), 77–114.

[B4] Asif M. J. Mak C. Othman R. Y. (2001). Characterization of indigenous *Musa* species based on flow cytometric analysis of ploidy and nuclear DNA content. Caryologia. 54 (2), 161–168. 10.1080/00087114.2001.10589223

[B5] Badaeva E. D. Amosova A. V. Goncharov N. P. Macas J. Ruban A. S. Grechishnikova I. V. (2015). A set of cytogenetic markers allows the precise identification of all A-genome chromosomes in diploid and polyploid wheat. Cytogenet. Genome Res. 146 (1), 71–79. 10.1159/000433458 26160023

[B6] Balint-Kurti P. Clendennen S. Doleželová M. Valárik M. Doležel J. Beetham P. R. (2000). Identification and chromosomal localization of the *monkey* retrotransposon in *Musa* sp. Mol. Gen. Genet. 263 (6), 908–915. 10.1007/s004380000265 10954075

[B7] Bartoš J. Alkhimova O. Doleželová M. De Langhe E. Doležel J. (2005). Nuclear genome size and genomic distribution of ribosomal DNA in *Musa* and *Ensete* (*Musaceae*): taxonomic implications. Cytogenet. Genome Res. 109, 50–57. 10.1159/000082381 15753558

[B8] Baurens F. C. Martin G. Hervouet C. Salmon F. Yohomé D. Ricci S. (2019). Recombination and large structural variations shape interspecific edible bananas genomes. Mol. Biol. Evol. 36 (1), 97–111. 10.1093/molbev/msy199 30403808 PMC6340459

[B9] Belser C. Istace B. Denis E. Dubarry M. Baurens F. C. Falentin C. (2018). Chromosome-scale assemblies of plant genomes using nanopore long reads and optical maps. Nat. Plants. 4 (11), 879–887. 10.1038/s41477-018-0289-4 30390080

[B10] Braz G. T. He L. Zhao H. Zhang T. Semrau K. Rouillard J. M. (2018). Comparative oligo-FISH mapping: An efficient and powerful methodology to reveal karyotypic and chromosomal evolution. Genetics. 208 (2), 513–523. 10.1534/genetics.117.300344 29242292 PMC5788518

[B11] Brown A. Tumuhimbise R. Amah D. Uwimana B. Nyine M. Mduma H. (2017). The genetic improvement of bananas and plantains (Musa spp.) In Genetic Improvement of Tropical Crops Vol. pp. Campos, HCaligari, PDS. Cham: Springer, 219–240.

[B12] Carreel F. Fauré S. González de León D. Lagoda P. J. L. Perrier X. Bakry F. (1994). Evaluation of the genetic diversity in diploid bananas (*Musa* sp.). Genet. Sel. Evol. 26 (Suppl 1), 125–136. 10.1051/gse:19940709

[B13] Cheesman E. E. (1947). Classification of the bananas. The genus *Ensete* Horan and the genus *Musa* L. Kew. Bull. 2 (2), 97–117. 10.2307/4109206

[B14] Čížková J. Hřibová E. Humplíková L. Christelová P. Suchánková P. Doležel J. (2013). Molecular analysis and genomic organization of major DNA satellites in banana (*Musa* spp.). PloS One 8, e54808. 10.1371/journal.pone.0054808 23372772 PMC3553004

[B15] Čížková J. Hřibová E. Christelová P. Van den Houwe I. Häkkinen M. Roux N. (2015). Molecular and cytogenetic characterization of wild *Musa* species. PLoS One 10:e0134096. 10.1371/journal.pone.0134096 26252482 PMC4529165

[B16] Cremer T. Cremer S. (2001). (B) Chromosome territories, nuclear architecture and gene regulation in mammalian cells.. Nat. Rev. Genet. 2 (4), 292–301. 10.1038/35066075 11283701

[B17] D’Hont A. Paget-Goy A. Escoute J. Carreel F. (2000). The interspecific genome structure of cultivated banana, *Musa* spp. revealed by genomic DNA *in situ* hybridization. Theor. Appl. Genet. 100, 177–183. 10.1007/s001220050024

[B18] D’Hont A. Denoeud F. Aury J. M. Baurens F. C. Carreel F. Garsmeur O. (2012). The banana (*Musa acuminata*) genome and the evolution of monocotyledonous plants. Nature 488, 213–217. 10.1038/nature11241 22801500

[B19] Danilova T. V. Friebe B. Gill B. S. (2014). Development of wheat single gene FISH map for analyzing homoeologous relationship and chromosomal rearrangements within the triticeae. Theor. Appl. Genet. 127 (3), 715–730. 10.1007/s00122-013-2253-z 24408375 PMC3931928

[B20] Davey M. W. Gudimella R. Harikrishna J. A. Sin L. W. Khalid N. Keulemans J. (2013). A draft *Musa balbisiana* genome sequence for molecular genetics in polyploid, inter- and intra-specific *Musa* hybrids. BMC Genomics 14, 683. 10.1186/1471-2164-14-683 24094114 PMC3852598

[B21] De Capdeville G. Souza Junior M. T. Szinay D. Diniz L. E. C. Wijnker E. Swennen R. (2009). The potential of high-resolution BAC-FISH in banana breeding. Euphytica 166, 431–443. 10.1007/s10681-008-9830-2

[B22] Doležel J. Doleželová M. Novák F. J. (1994). Flow cytometric estimation of nuclear DNA amount in diploid bananas (*Musa acuminata* and *Musa balbisiana*). Biol. Plant 36, 351–357. 10.1007/BF02920930

[B23] Doležel J. Doleželová M. Roux N. Van den houwe I. (1998). A novel method to prepare slides for high resolution chromosome studies in *Musa* spp. Infomusa 7, 3–4.

[B24] Doleželová M. Valárik M. Swennen R. Horry J. P. Doležel J. (1998). Physical mapping of the 18S-25S and 5S ribosomal RNA genes in diploid bananas. Biol. Plant 41, 497–505. 10.1023/A:1001880030275

[B25] Dupouy M. Baurens F. C. Derouault P. Hervouet C. Cardi C. Cruaud C. (2019). Two large reciprocal translocations characterized in the disease resistance-rich *burmannica* genetic group of *Musa acuminata*. Ann. Bot. XX, 1–11. 10.1093/aob/mcz078 PMC6758587 31241133

[B26] Ferguson-Smith M. A. Trifonov V. (2007). Mammalian karyotype evolution. Nat. Rev. Genet. 8 (12), 950–962. 10.1038/nrg2199 18007651

[B27] Filiault D. L. Ballerini E. S. Mandáková T. Aköz G. Derieg N. J. Schmutz J. (2018). The *Aquilegia* genome provides insight into adaptive radiation and reveals an extraordinarily polymorphic chromosome with a unique history. eLife 7, e36426. 10.7554/eLife.36426 30325307 PMC6255393

[B28] Fukui K. Kamisugi Y. Sakai F. (1994). Physical mapping of 5S rDNA loci by direct-cloned biotinylated probes in barley chromosomes. Genome. 37 (1), 105–111. 10.1139/g94-013 8181730

[B29] Gill B. S. Kimber G. (1977). Recognition of translocations and alien chromosome transfers in wheat by the Giemsa C-banding technique. Crop Sci. 17, 264–266. 10.2135/cropsci1977.0011183X001700020008x

[B30] Gill B. S. Friebe B. Endo T. R. (1991). Standard karyotype and nomenclature system for description of chromosome bands and structural aberrations in wheat (*Triticum aestivum*). Genome 34, 830–839. 10.1139/g91-128

[B31] Greilhuber J. (1977). Why plant chromosomes do not show G-bands. Theor. Appl. Genet. 50 (3), 121–124. 10.1007/BF00276805 24407608

[B32] Häkkinen M. (2013). Reappraisal of sectional taxonomy in *Musa* (*Musaceae*). Taxon. 62 (4), 809–813. 10.12705/624.3

[B33] Hamilton J. P. Buell C. R. (2012). Advances in plant genome sequencing. Plant J. 70 (1), 177–190. 10.1111/j.1365-313X.2012.04894.x 22449051

[B34] Han Y. Zhang T. Thammapichai P. Weng Y. Jiang J. (2015). Chromosome-specific painting in *Cucumis* species using bulked oligonucleotides. Genetics. 200, 771–779. 10.1534/genetics.115.177642 25971668 PMC4512542

[B35] He L. Braz G. T. Torres G. A. Jiang J. M. (2018). Chromosome painting in meiosis reveals pairing of specific chromosomes in polyploid *Solanum* species. Chromosoma 127, 505–513. 10.1007/s00412-018-0682-9 30242479

[B36] Hou L. Xu M. Zhang T. Xu Z. Wang W. Zhang J. (2018). BMC Plant Biol. 18 (1), 110. 10.1186/s12870-018-1325-2 29879904 PMC5991451

[B37] Hřibová E. Doleželová M. Town C. D. Macas J. Doležel J. (2007). Isolation and characterization of the highly repeated fraction of the banana genome. Cytogenet. Genome Res. 119 (3-4), 268–274. 10.1159/000112073 18253041

[B38] Hřibová E. Doleželová M. Doležel J. (2008). Localization of BAC clones on mitotic chromosomes of *Musa acuminata* using fluorescence *in situ* hybridization. Biol. Plant 52, 445–452. 10.1007/s10535-008-0089-1

[B39] Hřibová E. Neumann P. Matsumoto T. Roux N. Macas J. Doležel J. (2010). Repetitive part of the banana (*Musa acuminata*) genome investigated by low-depth 454 sequencing. BMC Plant Biol. 10, 204. 10.1186/1471-2229-10-204 20846365 PMC2956553

[B40] Idziak D. Hazuka I. Poliwczak B. Wiszynska A. Wolny E. Hasterok R. (2014). Insight into the karyotype evolution of *Brachypodium* species using comparative chromosome barcoding. PloS One 9 (3), e93503. 10.1371/journal.pone.0093503 24675822 PMC3968144

[B41] International Plant Genetic Resources Institute-International Network for the Improvement of Banana and Plantain/Centre de Coopération internationale en recherche agronomique pour le développement [IPGRI-INIBAP/CIRAD]International Plant Genetic Resources Institute-International Network for the Improvement of Banana and Plantain/Centre de Coopération internationale en recherche agronomique pour le développement [IPGRI-INIBAP/CIRAD] (1996). Description for Banana (*Musa* spp.). Int. Network for the Improvement of Banana and Plantain, Montpellier, France; Centre de coopération int. en recherche agronomique pour le développement, Montpellier, France; International Plant Genetic Resources Institute Press, Rome.

[B42] Janssens S. B. Vandelook F. De Langhe E. Verstraete B. Smets E. Van den Houwe I. (2016). Evolutionary dynamics and biogeography of *Musaceae* reveal a correlation between the diversification of the banana family and the geological and climatic history of Southeast Asia. New Phytol. 210 (4), 1453–1465. 10.1111/nph.13856 26832306 PMC5066818

[B43] Jiang J. Gill B. S. Wang G. L. Ronald P. C. Ward D. C. (1995). Metaphase and interphase fluorescence *in situ* hybridization mapping of the rice genome with bacterial artificial chromosomes. Proc. Natl. Acad. Sci. U. S. A. 92 (10), 4487–4491. 10.1073/pnas.92.10.4487 7753830 PMC41969

[B44] Jiang J. (2019). Fluorescence *in situ* hybridization in plants: recent developments and future applications. Chromosom. Res. 27 (3), 153–165. 10.1007/s10577-019-09607-z 30852707

[B45] Křivánková A. Kopecký D. Stočes Š. Doležel J. Hřibová E. (2017). Repetitive DNA: a versatile tool for karyotyping in *Festuca pratensis* Huds. Cytogenet. Genome Res. 151 (2), 96–105. 10.1159/000462915 28334706

[B46] Kamaté K. Brown S. Durand P. Bureau J. M. De Nay D. Trinh T. H. (2001). Nuclear DNA content and base composition in 28 taxa of *Musa*. Genome. 44, 622–627. 10.1139/g01-058 11550896

[B47] Kim J. S. Childs K. L. Islam-Faridi M. N. Menz M. A. Klein R. R. Klein P. E. (2002). Integrated karyotyping of sorghum by *in situ* hybridization of landed BACs. Genome. 45, 402–412. 10.1139/g01-141 11962637

[B48] Koo D. H. Zhao H. Jiang J. (2016). Chromatin-associated transcripts of tandemly repetitive DNA sequences revealed by RNA-FISH. Chromosome Res. 24 (4), 467–480. 10.1007/s10577-016-9537-5 27590598

[B49] Lapitan N. L. V. Brown S. E. Kennard W. Stephens J. L. Knudson D. L. (1997). FISH physical mapping with barley BAC clones. Plant J. 11, 149–156. 10.1046/j.1365313X.1997.11010149.x

[B50] Li L. F. Häkkinen M. Yuan Y. M. Hao G. Ge X. J. (2010). Molecular phylogeny and systematics of the banana family (*Musaceae*) inferred from multiple nuclear and chloroplast DNA fragments, with a special reference to the genus *Musa*. Mol. Phylogenet. Evol. 57 (1), 1–10. 10.1016/j.ympev.2010.06.021 20601004

[B51] Liu W. Rouse M. Friebe B. Jin Y. Gill B. Pumphrey M. O. (2011). Discovery and molecular mapping of a new gene conferring resistance to stem rust, Sr53, derived from *Aegilops geniculata* and characterization of spontaneous translocation stocks with reduced alien chromatin. Chromosome Res. 19 (5), 669–682. 10.1007/s10577-011-9226-3 21728140

[B52] Lysák M. A. Doleželová M. Horry J. P. Swennen R. Doležel J. (1999). Flow cytometric analysis of nuclear DNA content in *Musa*. Theor. Appl. Genet. 98 (8), 1344–1350. 10.1007/s001220051201

[B53] Lysák M. A. Fransz P. F. Ali H. B. M. Schubert I. (2001). Chromosome painting in *A. thaliana*. Plant J. 28, 689–697. 10.1046/j.1365-313x.2001.01194.x 11851915

[B54] Mandáková T. Lysák M. A. (2008). Chromosomal phylogeny and karyotype evolution in x = 7 crucifer species (*Brassicaceae*). Plant Cell 20, 2559–2570. 10.1105/tpc.108.062166 18836039 PMC2590746

[B55] Mandáková T. Marhold K. Lysák M. A. (2013). The widespread crucifer species *Cardamine flexulosa* is an allotetraploid with a conserved subgenomic structure. New Phytol. 201, 982–992. 10.1111/nph.12567 24400905

[B56] Martin G. Baurens F. C. Droc G. Rouard M. Cenci A. Kilian A. (2016). Improvement of the banana “*Musa acuminata*“ reference sequence using NGS data and semi-automated bioinformatics methods. BMC Genomics 17, 1–12. 10.1186/s12864-016-2579-4 26984673 PMC4793746

[B57] Meng Z. Zhang Z. L. Yan T. Y. Lin Q. F. Wang Y. Huang W. Y. (2018). Comprehensively characterizing the cytological features of *Saccharum spontaneum* by the development of a complete set of chromosome-specific oligo probes. Front. Plant Sci. 9, 1624. 10.3389/fpls.2018.01624 30459801 PMC6232525

[B58] Murata M. Heslop-Harrison J. S. Motoyoshi F. (1997). Physical mapping of the 5S ribosomal RNA genes in *Arabidopsis thaliana* by multi-color fluorescence *in situ* hybridization with cosmid clones. Plant J. 12 (1), 31–37. 10.1046/j.1365-313X.1997.12010031.x 9263450

[B59] Murgha Y. E. Rouillard J. M. Gulari E. (2014). Methods for the preparation of large quantities of complex single-stranded oligonucleotide libraries. PloS One 9, e94752. 10.1371/journal.pone.0094752 24733454 PMC3986247

[B60] Němečková A. Christelová P. Čížková J. Nyine M. Van den houwe I. Svačina R. (2018). Molecular and cytogenetic study of East African Highland Banana. Front. Plant Sci. 9, 1371. 10.3389/fpls.2018.01371 30337933 PMC6180188

[B61] Neumann P. Navrátilová A. Koblížková A. Kejnovský E. Hřibová E. Hobza R. (2011). Plant cytogenetic perspective. Mob. DNA 2 (1), 4. 10.1186/1759-8753-2-4 21371312 PMC3059260

[B62] Novák P. Hřibová E. Neumann P. Koblížková A. Doležel J. Macas J. (2014). Genome-wide analysis of repeat diversity across the family *Musaceae*. PloS One 9 (6), e98918. 10.1371/journal.pone.0098918 24932725 PMC4059648

[B63] Ortiz R. Swennen R. (2014). From crossbreeding to biotechnology-facilitated improvement of banana and plantain. Biotechnol. Adv. 32, 158–169. 10.1016/j.biotechadv.2013.09.010 24091289

[B64] Osuji J. O. Crouch J. Harrison G. Heslop-Harrison J. S. (1998). Molecular cytogenetics of *Musa* species, cultivars and hybrids: location of 18S-5.8S-25S and 5S rDNA and telomere-like sequences. Ann. Bot. 82, 243–248. 10.1006/anbo.1998.0674

[B65] Qu M. Li K. Han Y. Chen L. Li Z. Han Y. (2017). Integrated karyotyping of woodland strawberry (*Fragaria vesca*) with oligopaint FISH probes. Cytogenet. Genome Res. 153, 158–164. 10.1159/000485283 29262412

[B66] Said M. Hřibová E. Danilova T. V. Karafiátová M. Čížková J. Friebe B. (2018). The *Agropyron cristatum* karyotype, chromosome structure and cross-genome homoeology as revealed by fluorescence *in situ* hybridization with tandem repeats and wheat single-gene probes. Theor. Appl. Genet. 131 (10), 2213–2227. 10.1007/s00122-018-3148-9 30069594 PMC6154037

[B67] Schubert I. Fransz P. F. Fuchs J. De Jong J. H. (2001). Chromosome painting in plants. Methods Cell Sci. 23, 57–69. 10.1023/A:1013137415093 11741144

[B68] Simmonds N. W. Shepherd K. (1955). The taxonomy and origins of the cultivated bananas. J. Linn. Soc. Bot. 55, 302–312. 10.1111/j.1095-8339.1955.tb00015.x

[B69] Simmonds N. W. (1956). Botanical results of the banana collecting expeditions, 1954-5. Kew Bull. 11, 463–489. 10.2307/4109131

[B70] Speicher M. R. Ballard S. G. Ward D. C. (1996). Karyotyping human chromosomes by combinatorial multi-fluor FISH. Nat. Genet. 12 (4), 368–375. 10.1038/ng0496-368 8630489

[B71] The Arabidopsis Genome Initiative (2000). Analysis of the genome sequence of the flowering plant *Arabidopsis thaliana*. Nature 408, 6814, 796–815. 10.1038/35048692 11130711

[B72] The International Brachypodium Initiative (2010). Genome sequencing and analysis of the model grass *Brachypodium distachyon*. Nature 463 (7282), 763–768. 10.1038/nature08747 20148030

[B73] Valárik M. Šimková H. Hřibová E. Šafář J. Doleželová M. Doležel J. (2002). Isolation, characterization and chromosome localization of repetitive DNA sequences in bananas (*Musa* spp.). Chromosome Res. 10 (2), 89–100. 10.1023/A:1014945730035 11993938

[B74] Xin H. Zhang T. Han Y. Wu Y. Shi J. Xi M. (2018). Chromosome painting and comparative physical mapping of the sex chromosomes in *Populus tomentosa* and *Populus deltoides*. Chromosoma. 127, 313–321. 10.1007/s00412-018-0664-y 29520650

[B75] Zimin A. V. Puiu D. Hall R. Kingan S. Clavijo B. J. Salzberg S. L. (2017). The first near-complete assembly of the hexaploid bread wheat genome, *Triticum aestivum*. Gigascience. 6 (11), 1–7. 10.1093/gigascience/gix097 PMC5691383 29069494

