# Supplementary figures and images for: Chromosome Painting Facilitates Anchoring Reference Genome Sequence to Chromosomes In Situ and Integrated Karyotyping in Banana (Musa Spp.)
Source: Front Plant Sci. 2019 Nov 20;10:1503. doi: 10.3389/fpls.2019.01503 (PMC6879668; doi:10.3389/fpls.2019.01503)

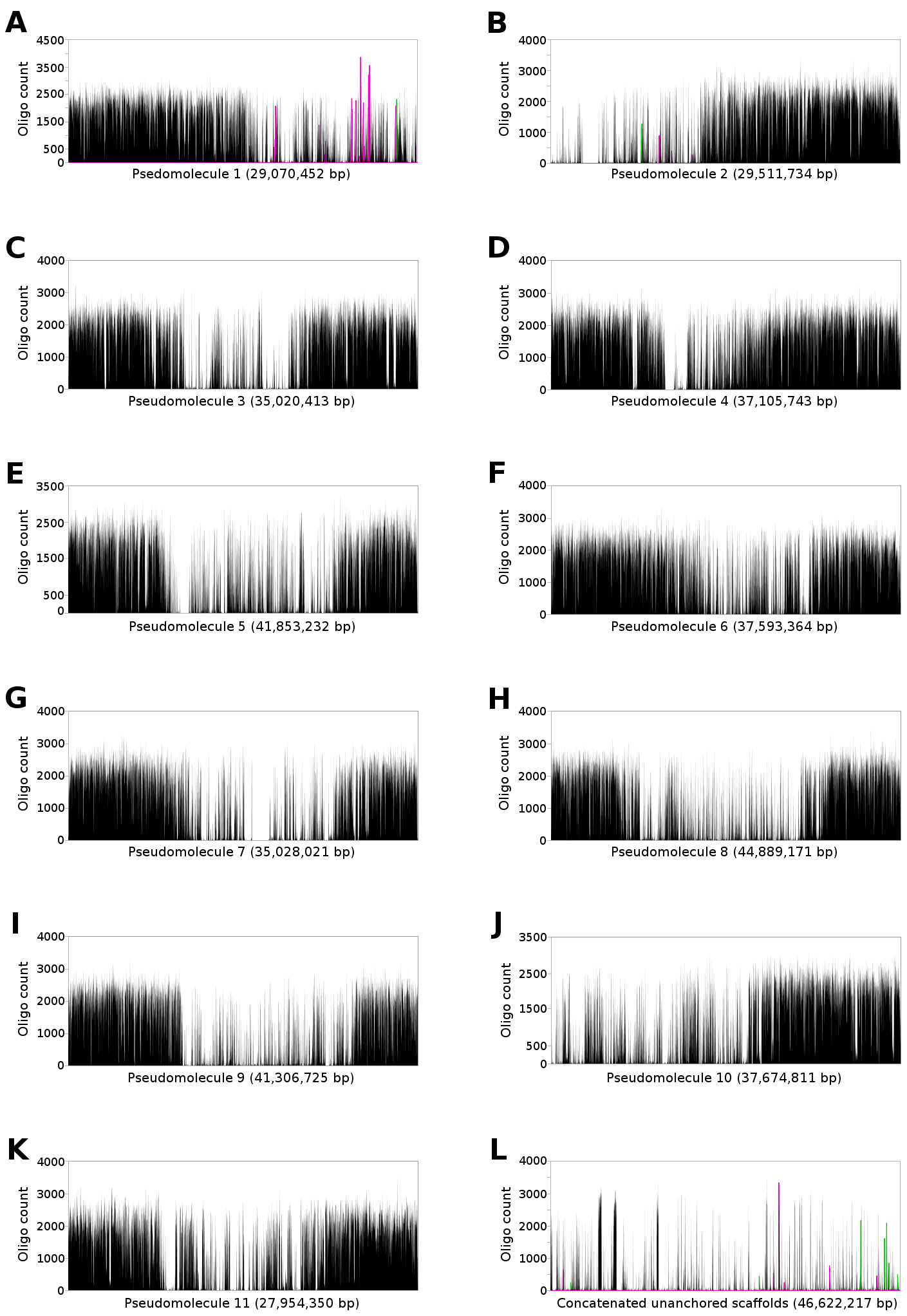

Supplement: Supplementary Figure S1 — Oligomer coverage of 11 pseudomolecules (labeled A– K) and concatenated unanchored scaffolds (L) in the reference genome of M. acuminata‘DH Pahang’ (Martin et al., 2016). The oligomers (45 bp) were designed using the Chorus program (Han et al., 2015) and are depicted in black. Position and coverage of tandem repeats CL18 (pink) and CL33 (green) are also shown. [file Image_1.tiff]
