# Supplementary material for: Chromosome Painting Facilitates Anchoring Reference Genome Sequence to Chromosomes In Situ and Integrated Karyotyping in Banana (Musa Spp.)
Source: Front Plant Sci. 2019 Nov 20;10:1503. doi: 10.3389/fpls.2019.01503 (PMC6879668; doi:10.3389/fpls.2019.01503)
Supplement: Supplementary file 2 [file Table_1.docx]

**Supplementary Table S1.** The size of painted regions and density of oligomers per 1 kb on pseudomolecules of *M. acuminata* ‘DH Pahang’ genome assembly v2 (Martin *et al*., 2016). In this assembly, pseudomolecules of chromosomes 1, 2 and 10 are represented only by long chromosome arms.

| **Chromosome / arm** | **Position on pseudomolecule [Mb]** | **Length of**  **painted region [kb]** | **Oligomer density**  **per 1 kb** |
| --- | --- | --- | --- |
| Chromosome 1L * | 1 – 15.050 | 15,050 | 1.3 |
| Chromosome 2L | 12.831 – 29.511 | 12,830 | 1.2 |
| Chromosome 3S | 1 – 11.646 | 11,640 | 1.6 |
| Chromosome 3L | 22.111 – 35.020 | 12,909 | 1.5 |
| Chromosome 4S | 1 – 12.100 | 12,100 | 1.6 |
| Chromosome 4L | 14.400 – 37.105 | 22,705 | 0.9 |
| Chromosome 5S | 1 – 12.305 | 12,385 | 1.5 |
| Chromosome 5L | 14.765 – 41.853 | 11,130 | 1.4 |
| Chromosome 6S * | 21.253 – 37.564 | 17,355 | 1.2 |
| Chromosome 6L * | 1 – 17.355 | 16,310 | 1.2 |
| Chromosome 7S * | 21.554 – 35.028 | 13,400 | 1.5 |
| Chromosome 7L * | 1 – 17.756 | 14,850 | 1.5 |
| Chromosome 8S | 1 – 18.805 | 18,805 | 1.7 |
| Chromosome 8L | 31.882 – 44.889 | 13,000 | 1.6 |
| Chromosome 9S | 1 – 13.514 | 13,510 | 1.4 |
| Chromosome 9L | 25.910 – 41.306 | 15,390 | 1.6 |
| Chromosome 10L | 21.028 – 37.674 | 16,340 | 0.9 |
| Chromosome 11S | 1 – 11.003 | 11,000 | 1.8 |
| Chromosome 11L | 15.635 – 27.954 | 12,310 | 2.1 |

* In the assembly, pseudomolecules 1, 6, 7 start with long arms and end with short arms, i.e. they are oriented inversely to the way karyotypes are presented, where the short arm of the chromosome is on top and the long arm on the bottom.
